# Machine Learning Approaches in Brillouin Distributed Fiber Optic Sensors

**DOI:** 10.3390/s23136187

**Published:** 2023-07-06

**Authors:** Christos Karapanagiotis, Katerina Krebber

**Affiliations:** Bundesanstalt für Materialforschung und-Prüfung, Unter den Eichen 87, 12205 Berlin, Germany; katerina.krebber@bam.de

**Keywords:** distributed fiber optic sensors, Brillouin scattering, BOTDA, BOFDA, machine learning, artificial neural networks, structural health monitoring, strain and temperature measurements

## Abstract

This paper presents reported machine learning approaches in the field of Brillouin distributed fiber optic sensors (DFOSs). The increasing popularity of Brillouin DFOSs stems from their capability to continuously monitor temperature and strain along kilometer-long optical fibers, rendering them attractive for industrial applications, such as the structural health monitoring of large civil infrastructures and pipelines. In recent years, machine learning has been integrated into the Brillouin DFOS signal processing, resulting in fast and enhanced temperature, strain, and humidity measurements without increasing the system’s cost. Machine learning has also contributed to enhanced spatial resolution in Brillouin optical time domain analysis (BOTDA) systems and shorter measurement times in Brillouin optical frequency domain analysis (BOFDA) systems. This paper provides an overview of the applied machine learning methodologies in Brillouin DFOSs, as well as future perspectives in this area.

## 1. Introduction

Over the last few years, machine learning has revealed the untapped potential for advanced signal processing and provided new avenues for innovation and progress in the field of distributed fiber optic sensors (DFOSs). DFOSs enable continuous measurements along the entire length of an optical fiber, which can be up to hundreds of kilometers. This has already made DFOSs attractive for a wide range of applications, including structural health monitoring of civil and geotechnical structures [1,2,3,4,5], pipeline and borehole monitoring for leak detection [6], seismic activity monitoring [7,8,9,10] or even the condition monitoring of high-voltage submarine cables [11] and deep earth dynamics in oceans [12]. Even though the most common measurands are temperature and strain, DFOSs can directly or indirectly measure the humidity [13,14,15], pressure [16], displacement [4,17], radiation [18,19,20], gas concentration [21,22], etc.

DFOSs are primarily categorized based on the scattering mechanisms, which can be Rayleigh, Brillouin or Raman [23]. Rayleigh-based DFOSs rely on the detection of the backscattered light generated by the interaction between the light and the fiber’s inherent refractive index fluctuations. This technique provides the strongest signal and is ideal for dynamic sensing applications, such as distributed acoustic sensing (DAS). Rayleigh-based DFOSs do not require signal averaging and can provide real-time monitoring. For the sake of completeness, we mention that many Rayleigh-based DFOSs operating either in the time or frequency domain have been developed and proposed. Similar to Brillouin-based DFOSs, these sensors can be used for temperature and strain monitoring [24,25,26,27]. Brillouin-based DFOSs rely on the detection of the Brillouin scattering generated by the interaction between the light and the acoustic waves propagating along the fiber. This technique is highly sensitive and provides accurate measurements of temperature and strain. However, Brillouin scattering is relatively weak in comparison to Rayleigh scattering, and signal averaging is typically required to obtain signals of a high signal-to-noise ratio (SNR). Therefore, a Brillouin-based DFOS is better suited for static or quasi-static monitoring applications where the changes occur over longer periods and real-time monitoring is not required. Nonetheless, it is worth noting that solutions for dynamic Brillouin DFOSs have also been proposed [28,29,30,31].

Brillouin DFOSs are typically classified into two main categories: time domain and frequency domain systems. Both techniques offer long measurement ranges and high spatial resolution [32]. Specifically, measurement lengths of up to 200 km [33,34,35] and spatial resolutions even on centimeter or millimeter scales [36,37,38] have been reported. Time domain systems, such as Brillouin optical time domain analyzers (BOTDAs), directly measure the pulse response, while the frequency domain systems, such as Brillouin optical frequency domain analyzers (BOFDAs) retrieve the pulse response by applying inverse fast Fourier transformation to the measured complex transfer function [39]. Regardless, time domain analysis is significantly faster than frequency domain analysis; the latter does not necessitate the use of fast sampling circuits, which positively affects the system’s cost [32,39].

The emergence of machine learning methodologies in DFOSs has been driven by several important factors. First, state-of-the-art DFOS systems allow for continuous and long-range monitoring, generating massive amounts of data that are difficult and time-consuming to analyze manually [40,41,42,43]. This creates opportunities for advanced signal processing and analysis using machine learning techniques, which can effectively extract meaningful insights from the vast amounts of data generated by the DFOS. Second, recent progress in big data and cloud technologies provides tools for the efficient storage and processing of large volumes of data. Third, the significant progress and successful application of machine learning in various fields prior to its use in DFOSs motivated and facilitated the adoption of machine learning techniques to DFOSs. Last, the development of powerful graphical processing units (GPUs) enabled fast and advanced machine learning analysis.

DAS allows for continuous and real-time monitoring, which can result in enormous amounts of data over time. This motivated the use of machine learning, which has been used to process big amounts of data in order to detect and classify events or damages [44,45,46]. Specifically, artificial neural networks (ANNs) have been proposed for classifying external intrusion signals to increase safety in oil and gasoline pipelines [47,48,49,50,51,52,53]. Furthermore, machine learning has been also proposed in DFOSs for monitoring railway tracks and trains and detecting patterns and anomalies that could indicate potential issues [54,55,56,57]. Apart from infrastructure condition monitoring, deep neural networks have also been proposed to accurately detect earthquakes from data collected by DAS [58]. In addition to these specific applications, machine learning has also enabled significant advances in DAS systems that are independent of the application. As an example, machine learning algorithms have been used to denoise signals [59,60,61,62] faster than conventional denoising algorithms, allowing for extended measurement lengths [63], and have replaced less efficient signal processing algorithms, such as cross-correlation [64,65]. For the sake of completeness, we note that machine learning has also been applied.

Machine learning in Brillouin DFOSs has been applied in various stages of the signal processing. Specifically, machine learning algorithms have been employed to enhance the measurement accuracy and shorten the signal processing time without increasing the system’s cost [40,66,67]. Machine learning has also contributed to enhanced spatial resolution in BOTDA systems [68] and shorter measurement times in BOFDA systems [69]. Furthermore, the problem of temperature and strain cross-sensitivity has been addressed using machine learning in both BOTDA and BOFDA systems [70,71,72,73]. As we discuss later in Section 2, the decoupling of temperature and strain effects has also been achieved using methods, including a two-fiber configuration [74], hybrid systems employing more than one scattering effect [75,76,77,78], and specialty fibers [79,80,81,82,83]. However, machine learning does not increase the system’s cost or hardware complexity and can be applied even in standard telecom optical fibers [70].

The aim of this paper is to succinctly present a concise overview and comparison of the machine learning approaches reported in Brillouin DFOSs. Furthermore, we identify the challenges associated with these approaches and suggest areas for further investigations in the future. The paper is structured as follows: after this introduction, we present the most-known types of Brillouin DFOS systems and describe the basic signal processing methods. The third section of the paper describes the machine learning methodologies that have been applied mostly in time domain systems to enhance temperature and strain accuracy. The first part of this section compares machine learning methodologies applied for Brillouin frequency shift (BFS) extraction, which is the most conventional feature for estimating temperature and strain changes. The second part of the section provides an overview of the machine learning-based denoising methods and compares them with others employed mostly in the field of image processing. The third part discusses machine learning approaches for temperature and strain extraction directly from the Brillouin gain spectrum (BGS) without feature extraction, such as BFS. The fourth section presents machine learning methodologies applied in BOFDA sensors for shortening the measurement time and measuring simultaneous temperature and strain, as well as temperature and humidity.

## 2. Brillouin Distributed Fiber Optic Sensors (DFOSs)

**Figure 1:**
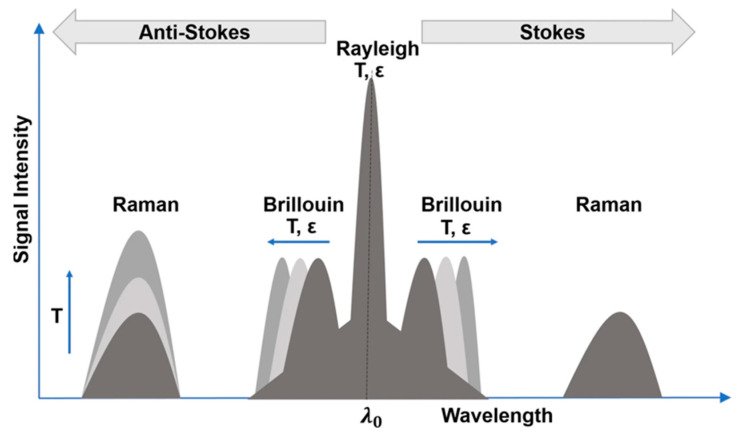
Schematic representation of the Rayleigh, Brillouin and Raman scattering effects in optical fibers providing a rough estimation of the backscattered intensity or frequency changes with temperature or strain. Copyright 2021 licensed under a Creative Commons Attribution 4.0 International license [84].

In this section, we describe the most-known types of Brillouin DFOS systems and the conventional signal processing for temperature or strain extraction. Rayleigh scattering is elastic and arises from the non-propagating density fluctuations of the medium. Because this scattering effect is the strongest, no signal averaging is needed, and thus, Rayleigh DFOSs are widely used for vibration monitoring. Brillouin and Raman scattering effects are inelastic and originate from the interaction of the propagating light with the acoustic and optical phonons, respectively. Furthermore, the frequency downshifted and upshifted components resulting from these interactions are called “Stokes” and “anti-Stokes”, respectively. Raman DFOSs are mainly used for temperature sensing, while Brillouin DFOSs provide temperature and strain information. We note that in Brillouin DFOS, the temperature and strain information is related to the frequency difference between the incident and the scattered Stokes or Anti-stokes light. This frequency difference is called the Brillouin frequency shift (BFS). A schematic representation of the scattering effects is shown in Figure 1.

**Figure 2:**
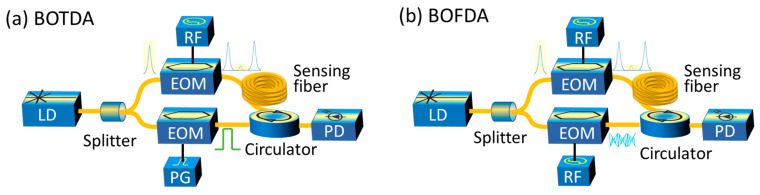
Two most common types of Brillouin-distributed fiber optic sensors based on the time (**a**) and frequency (**b**) domain. BOTDA: Brillouin optical time domain analysis; BOFDA: Brillouin optical frequency domain analysis; EOM: electro-optic modulator; RF: radio frequency; PG: pulse generator; PD: photodiode.

Three well-known types of Brillouin DFOSs that are reported in the literature are the time domain, frequency domain and correlation (coherence) domain systems [23]. Time domain approaches make use of pulses that travel down the fiber and get scattered and finally detected by a photodiode. The recorded pulse response over time can be converted into a spatially resolved gain profile, providing that the refractive index of the medium is known. On the other hand, the frequency domain systems make use of RF-modulated continuous waves and measure the system’s complex transfer function [39]. The complex function can in turn be converted into the time domain through inverse fast Fourier transformations. The interrogation approach in correlation domain systems differs significantly from the previous approaches. The correlation domain technique is position-selective, which allows for measurements even at small regions of the optical fiber and offers enhanced spatial resolution [85,86,87]. The position to be measured is determined based on the interference characteristics of two RF-modulated continuous waves. Furthermore, we note that all the aforementioned techniques can be implemented using the single-end or the double-end configuration. The difference between these two configurations is that the first is based on spontaneous Brillouin scattering, while the second is based on stimulated Brillouin scattering. The stimulated scattering requires an additional continuous counterpropagating wave with a frequency equal to the spontaneous Brillouin scattered wave. The frequency tunning of the counterpropagating wave is performed by an EOM which is driven by an RF signal generator. Even though the double-end configuration requires access to both ends of the fiber, the signal is stronger than that obtained by the single-end systems [23]. The time, frequency and correlation domain systems based on the double-end configurations are conventionally called Brillouin optical time domain analysis (BOTDA), Brillouin optical frequency domain analysis (BOFDA) and Brillouin optical correlation domain analysis (BOCDA), respectively. If only the end of the fiber is used, then the system that works, for example, in time domain, is called Brillouin optical time domain reflectometry (BOTDR). Figure 2 provides a schematic of the most common Brillouin DFOS systems, including only some basic key components.

Even though the data acquisition process differs from system to system, the signal processing for temperature and strain extraction from the so-called Brillouin gain spectrum (BGS) is similar. The most conventional feature is the Brillouin frequency shift (BFS), which is extracted by performing Lorentzian curve fitting (LCF) on the BGS data. We note that, apart from Lorentzian curves, Gaussian or pseudo-Voigt curves have also been employed and in some cases delivered a more accurate BFS [88,89]. Furthermore, BFS extraction based on cross-correlation is also common in the literature [90,91]. The BFS depends linearly on temperature and strain, and thus, the temperature or strain change can be estimated, providing that the temperature and strain coefficients are known. These coefficients are unique for every fiber, and unless they are provided by the manufacturer, their estimation requires a preliminary analysis of the BFS under different temperature and strain conditions.

Simultaneous measurements of temperature and strain are not trivial due to the cross-sensitivity effects. This means that changes in one parameter can be measured as long as the other one is constant. This problem has been addressed by using two optical fibers, placed in parallel and close to each other with the one being mechanically isolated [74]. However, the two-fiber configuration is impractical for many applications. Temperature and strain discrimination has been demonstrated using hybrid systems employing more than one scattering effect or specialty fibers [75,76,77,78]. Some specialty fibers, such as large effective area fibers (LEAFs) [79,80,81], photonic crystal fibers [82], and dispersion compensating fibers [83] offer a multipeak BGS with at least two Brillouin peaks, with different temperature and strain sensitivities. In that case, one extracts simultaneously the temperature (T) and strain (ε) by solving a system of equations, as follows:

(1) $$BFS^{peak1}=C_{T}^{peak1}T+C_{\varepsilon }^{peak1}\varepsilon$$

(2) $$BFS^{peak2}=C_{T}^{peak2}T+C_{\varepsilon }^{peak2}\varepsilon$$

where $C_{T}$ and $C_{\varepsilon }$ are the temperature and strain coefficients, respectively.

## 3. Machine Learning Applied in Brillouin Time Domain Sensors

The conventional signal processing can be cumbersome, especially when the SNR is relatively low. When the data are noisy, the Lorentzian fitting optimization is significantly slower, and erroneous estimations of the BFS are expected. Machine learning has been proposed to partly or completely replace the conventional signal processing methods. Specifically, machine learning has been utilized to accelerate the BFS extraction, denoise the BGS, enable fast temperature extraction directly from the BGS and discriminate temperature and strain effects.

### 3.1. Machine Learning for Feature Extraction from the Brillouin Gain Spectrum

Many types of machine learning were proposed to extract the BFS. The LCF can be cumbersome, especially in cases with low SNR, which in turn, results in slow and inaccurate temperature or strain estimations. Machine learning was applied to overcome these limitations and provide a more efficient way to extract the BFS leading to more accurate and faster temperature or strain measurements. To this end, many types of machine learning algorithms were proposed, including artificial neural networks (ANNs), convolutional neural networks (CNNs), support vector machines (SVMs), k-nearest neighbors (KNNs), etc.

**Figure 3:**
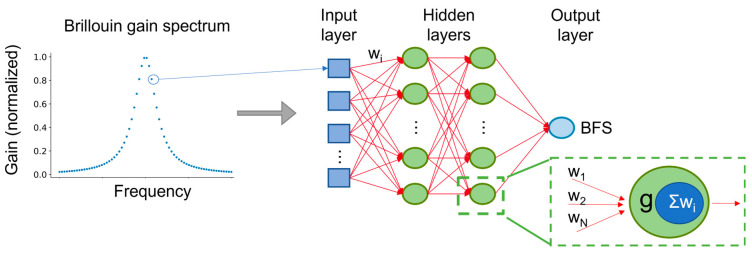
Brillouin frequency shift (BFS) extraction using an artificial neural network (ANN). w: weight; Σw: weighted sum; g: activation function.

Figure 3 shows a schematic of the ANN methodology for BFS extraction reported by Liang et al. [92]. Instead of performing LCF on the data points of the BGS, those data points were given as inputs to an ANN. The proposed ANN consisted of two hidden layers. The hidden layers of the ANNs consist of nodes that are nothing more than activation functions applied to the weighted sums of the outputs of all the nodes of the previous layer. The ANN training aims at optimizing the weights so that the error of the output is minimized. The optimization algorithm is based on backpropagation [93]. Liang et al. [92] trained an ANN and evaluated its performance using synthetic and experimental data, respectively. To increase the model’s robustness, the training dataset included different frequency ranges, linewidths and noise levels. The authors note that both the inputs and the outputs were normalized before training. The normalization of the input and the output facilitates the model generalization based on the BGS with different gains and different scanning frequencies, respectively.

The optimization of the hyperparameters (number of hidden layers, number of nodes, type of activation function, etc.) is of great importance in all machine learning models. Liang et al. [92] used a validation dataset to optimize the ANN during training and applied early stopping to avoid overfitting. For the sake of completeness, we mention that overfitting refers to the model’s failure to generalize based on new data [94], while early stopping stops the training procedure when the model’s performance based on the validation dataset starts degrading [95]. We note that the complexity of the ANN architectures is strongly related to the prediction times. Therefore, the relatively simple architecture proposed by Liang et al. [92] proved to be very fast. Specifically, the final ANN model required approximately only 1.2 s to process 100,000 BGSs. Even though ANNs can deliver fast predictions, the training time is usually time-consuming. In this case, the reported training time was approximately three hours. Furthermore, the authors tested the final ANN model based on real experimentally obtained data using a BOTDA system. The BFS errors were found to be very close to the LCF errors.

**Figure 4:**
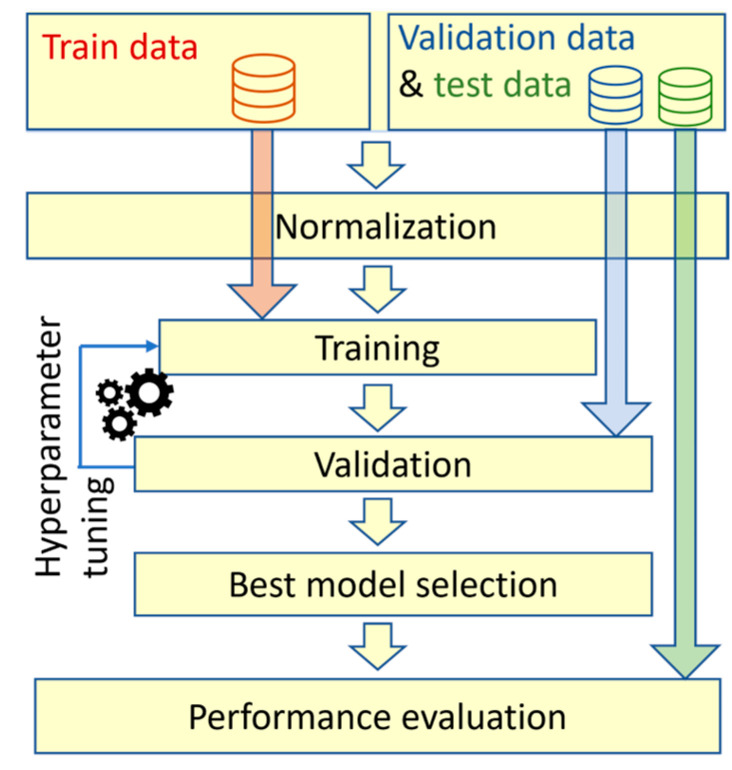
Common training procedure in machine learning.

The described training and model’s evaluation procedure is shown in Figure 4. This training pipeline is very common in machine learning and has been used in the majority of the papers that are discussed here. The train and validation data usually consist of synthetic data, while the test data result from lab or field experiments. Before training and testing, all data are normalized. During the training process, the algorithm undergoes multiple iterations (epochs) based on the training dataset. After each epoch, the model’s performance is evaluated by assessing its ability to generalize based on the validation dataset. This training procedure is repeated many times with different hyperparameter settings. This hyperparameter tuning process is a common practice in machine learning, as it helps to find the most effective settings for the algorithm. The final model is selected based on the performance using the validation dataset. Finally, to assess the overall effectiveness of the trained model, it is evaluated using a separate and independent dataset called the test data. This step provides an unbiased measure of the model’s performance based on unseen data, confirming its generalization capabilities.

We note that apart from the described training pipeline, methods based on cross-validation are also used, especially when the datasets are limited. Specifically, cross-validation is based on data resampling and repeatedly splits the dataset into train and validation sets. This technique has been widely applied in machine learning providing an unbiased estimation of the model’s performance [96].

In a more recent paper, Liang et al. [97] improved the ANN model to deal with a distorted BGS, caused by nonlocal effects. BGSs with nonlocal effects were simulated to acquire a new training dataset. The new ANN model resulted in significantly reduced BFS errors, although the network’s architecture changed only slightly (minus 10 and 5 nodes in the first and second layer, respectively). In comparison to the previous ANN and the conventional LCF method, an at least a five-fold reduction in the estimated BFS errors is reported. These results highlight the importance of the dataset in machine learning applications.

**Figure 5:**
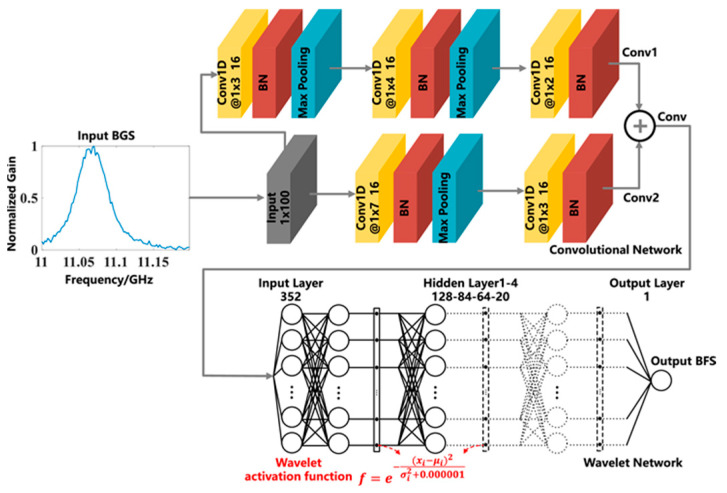
Architecture of the “wavelet” convolutional neural network (CNN) consisting of two paths of convolutional layers (**top**) and a stack of fully connected wavelet layers (**bottom**). One-dimensional convolutional layer (Conv 1D); Batch normalization (BN). Reprinted with permission from [98] © Optica Publishing Group.

Recently, Chen et al. [98] proposed one-dimensional CNNs for BFS extraction and compared their approach with the conventional LCF and the simple ANN. Specifically, the authors used a special type of CNN, called wavelet convolutional neural network. The architecture of the proposed network is shown in Figure 5. It consists of two paths of convolutional layers, which end up in a fully connected neural network after a residual connection is applied. The term “wavelet” arises from the type of activation function that is used in the fully connected network. The authors assert that the wavelet activation function was employed to cover more local characteristics in the frequency domain. The input of the CNN is a single normalized BGS consisting of 100 frequency scanning points, while the output is a single value indicating the BFS. The batch normalization and max pooling layers are used to address the covariance shift problem and to down-sample the data, respectively.

**Figure 6:**
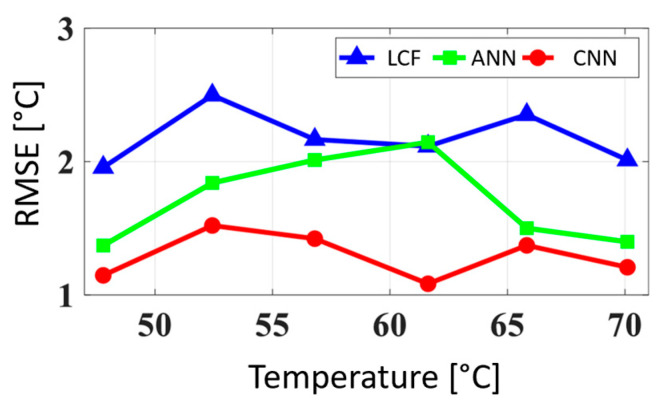
The root mean square error (RMSE) of the extracted temperature using Lorentzian curve fitting (LCF), artificial neural networks (ANN) and convolutional neural networks (CNN), adapted with permission from [98] © Optica Publishing Group.

Similar to the previous methodology, the authors made use of synthetic data for training. The data consisted of different BFSs, linewidths and SNRs. The model’s evaluation based on experimental data, obtained by a BOTDR system, showed an improvement in terms of temperature error in comparison to the conventional LCF and a simple ANN consisting of two hidden layers. Specifically, the results indicated that the temperature root mean square error (RMSE) of the CNN is approximately 1 °C lower than that of the conventional LCF method. However, the improvement of the CNN in comparison to the ANN seems to highly depend on the temperature. For example, the error difference at 61.62 °C is around 1 °C, while at 65.82 °C, it becomes negligible. The results are shown in Figure 6. We note that the authors trained the ANN and CNN using the same hardware and software.

**Figure 7:**
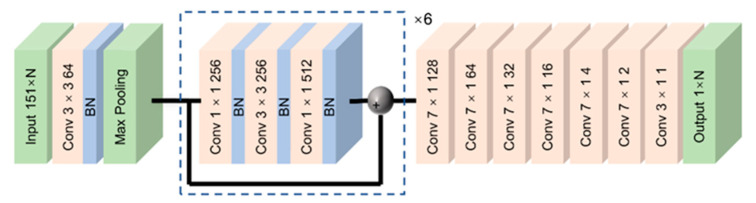
Convolutional neural network (CNN) for distributed Brillouin frequency shift (BFS) extraction. Reprinted with permission from [99] © Chinese Laser Press.

Chang et al. [99] reported that due to the correlation of the BGS in the time domain, a two-dimensional (2D) CNN that extracts distributed the BFS directly from distributed BGSs could be advantageous. Specifically, they demonstrated a CNN architecture, as shown in Figure 7, which consists of a 2D convolutional layer, a batch normalization layer and a single max pooling layer. After the max pooling layer, which reduces the dimensions of the processed data, a residual subnetwork consisting of a series of convolutional and batch normalization layers, is placed. The authors claimed that the use of that subnetwork facilitates the feature perception in the time and frequency domain as well. The last part of the CNN consists of consecutive 2D convolutional layers with a decreasing number of filters. In contrast to the CNN architecture in Figure 5, this CNN does not include fully connected layers. The size of the input layer, 151 × N, refers to the number of data points of the BGS and the number of distributed BGSs, respectively.

Similar to the previous methods, both the inputs and the outputs were normalized. The training set arose from synthetic data, including the BGS with different BFSs, linewidths and SNR values. The reported training time was approximately two hours using an Nvidia GTX 1080 GPU. It is notable that in comparison to a CPU, a GPU results in significantly faster training times [100].

The performance evaluation based on experimental data, collected with a BOTDA system, showed that in comparison to the conventional LCF method, the CNN has slightly improved the error of the BFS estimation. However, the authors are confident that the performance could be further improved by optimizing the CNN architecture and the training dataset. Furthermore, the authors reported that the CNN required only 0.13 s for the processing of 1000 BGSs, while the corresponding computation time for the conventional LCF approach was 0.81 s. A similar speed enhancement was also reported by Qi et al. [101].

**Figure 8:**
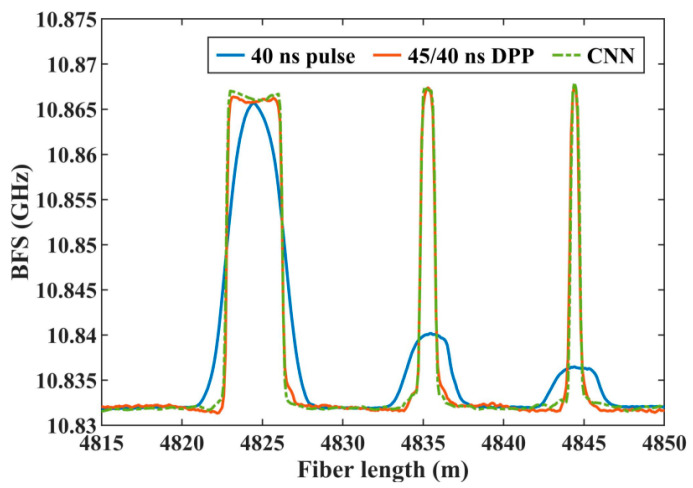
Brillouin frequency shift (BFS) estimation using a conventional BOTDA, a DPP-BOTDA and a CNN-BOTDA. Reprinted with permission from [68] © IEEE.

Ge et al. [68] showed that similar 2D CNNs can also result in enhanced spatial resolution in BOTDA and particularly when long pulses are used. Long pulses in BOTDA result in longer measurement lengths but on the other hand, decrease the spatial resolution. Conventionally, this trade-off problem can be alleviated by implementing a differential pulse-width pair (DPP), but at the cost of a two-fold increase in measurement time. Ge et al. [68] showed that a CNN-assisted BOTDA is capable of reaching the resolution of the DPP-BOTDA without increasing the measurement time. An example of the BFS estimation accuracy is shown in Figure 8. Caceres et al. [102] used similar CNNs to enhance the spatial resolution in BOCDR/BOCDA sensors.

**Figure 9:**
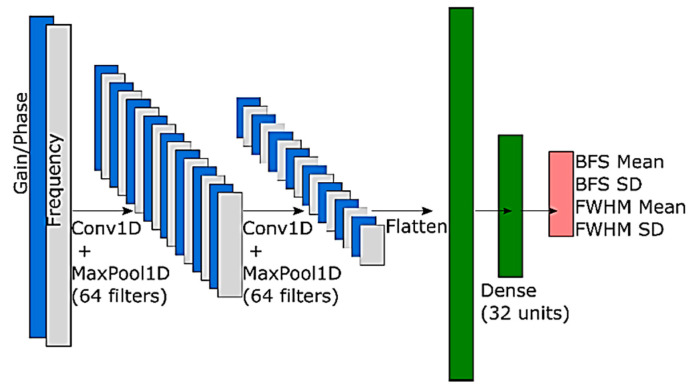
Probabilistic convolutional neural network for Brillouin frequency shift and linewidth extraction. Reprinted with permission from [103] © SPIE.

Lalam et al. [103] aimed at increasing the reliability of the neural networks. They proposed probabilistic neural networks that provide not only a point estimate of the BFS but also the prediction’s uncertainty, which is a measure to assess the model’s confidence. Therefore, when the model’s prediction is not precise enough, this is indicated by the provided uncertainty. Furthermore, the neural network outputs the full width at half maximum (FWHM) of the Lorentzian curve as well. The structure is shown in Figure 9. For the sake of completeness, we note that BFS uncertainties were also extracted using LCF and classic [104] or Bayesian statistics [105].

Apart from neural networks, simpler machine learning methods, including SVM, AdaBoost and KNN, have been applied for BFS extraction. SVMs are supervised learning models that have been widely used in classification and regression analysis [93]. In contrast to ANNs that require a big amount of data, SVM proved very efficient even if the available dataset is limited [106]. SVMs separate classes by constructing hyperplanes (decision surfaces) in high-dimensional spaces. SVM is named after the so-called support vectors, which are the data points that determine the orientation and position of the hyperplanes. Furthermore, SVM is based on kernels, which can be specified by e.g., linear, polynomial and radial basis functions [106]. Yao et al. [107] compared the influence of different kernel functions on the BFS estimation and found that the Gaussian radial basis function delivers the lowest errors. However, the width of the Gaussian kernel needs to be optimized so that overfitting is addressed. Yao et al. [107] also commented on the training speed of the SVM, which in general, is shorter than that of the ANNs. Specifically, the authors mentioned that the training of the SVM lasted only several minutes, which is a significant advantage over the ANN.

Zheng et al. [108] applied AdaBoost to extract the FBS. The AdaBoost algorithm trains many weak classifiers, which are weighted depending on the classification rate that they provide [109,110]. In the end, a strong classifier consisting of many weak classifiers arises. The weak classifiers that the authors chose were simple decision trees. The authors claimed that in cases of low SNR, where the LCF fails, the AdaBoost predicts the BFS with relatively low errors (approximately 1 MHz). However, no information was provided about the training and the prediction times. Furthermore, the trained AdaBoost is a classifier, which means that no interpolation is possible. We believe that this problem could be addressed by applying linear decision trees for regression [111,112].

In contrast to the previous algorithms, KNNs do not learn any model, and thus, no training is needed [113]. This is a great advantage over other algorithms that require time-consuming training (such as ANN and CNN). However, a dataset, including a plethora of BGSs and BFSs is required because the KNN predictions are based on feature similarity. Furthermore, the KNNs are characterized by two hyperparameters, namely the distance function and the number of neighbors (k-value) to be considered. Zheng et al. [114,115] made use of the Euclidean distance and optimized the k-value after a systematic analysis of its impact on the BFS extraction. The results based on experimental data showed that the KNNs provide lower BFS errors than those from the conventional LCF approach but only if the SNR is low. This indicates that KNNs are more tolerant against noise than the LCF.

Even though the proposed machine learning algorithms for BFS extraction have proved very efficient, the requirement for fixed input dimensions is a significant limitation. It is known that machine learning algorithms, in general, make predictions only based on data with the same dimensions as the data that were provided to the algorithm during training. This is of course impractical because the number of scanning frequencies, as well as the frequency range, can vary depending on the application. To address this issue, Liang et al. [92] applied linear interpolation based on the BGS so that the BGS always consists of the same number of frequencies before it is processed by the machine learning model. Furthermore, Xiao et al. [116] and Yao et al. [107] addressed this issue by regulating the input dimensions with principal component analysis (PCA). Apart from this, PCA also had a positive impact on the training time. We note that PCA is commonly used in data analysis to reduce the dimensions of the data without losing significant information [117].

Among the most common weaknesses in machine learning is the long training times that are related to the complexity of the algorithms. Usually, the more complex the algorithm, the longer the training. ANN and CNN are considered very complex, and usually, the training lasts several hours. Considering also the optimization of the hyperparameters, the total training time increases dramatically. This could be addressed to some extent using simpler architectures and state-of-the-art optimization techniques [118,119,120].

Interpretability is of great importance for every machine learning algorithm. Although some simple algorithms, such as linear and polynomial regression, are considered interpretable by themselves, ANNs and CNNs are usually treated as black boxes. This arises from their complexity, which renders the interpretation of their decisions very difficult. However, in the last few years, interpretable machine learning has gained much attention and has already made significant progress. As an example, we mention that sensitivity analysis, Taylor decomposition, deconvolution, guided backpropagation and layer-wise relevance propagation are among the state-of-the-art techniques that have been proposed to shed light on the neural networks’ decisions [121]. Other algorithms, such as KNN, SVM and AdaBoost (decision trees), are easier to interpret. We note that in comparison to all the aforementioned machine learning algorithms, KNN offers the fastest and easiest interpretation [122]. We believe that further research on the interpretation of the proposed machine learning algorithms for BFS extraction will create more trust, contribute to a more efficient hyperparameter optimization and open the way for wider use in the future.

### 3.2. Machine Learning for Denoising the Brillouin Gain Spectrum

Denoising techniques have resulted in enhanced BFSs and temperature accuracy in Brillouin DFOSs when the SNR is low. Low SNR usually arises either from short measurements, including only a few signal averages, or from distant positions in long optical fibers. However, conventional denoising methods are based, in general, on time-consuming optimization algorithms. For this reason, neural network-based denoising methods have been proposed. Although the training of such algorithms is usually time-consuming, the denoising process is very fast. This is attributed to the fact that, once a neural network model is trained, the predictions themselves do not include any optimization task.

**Figure 10:**
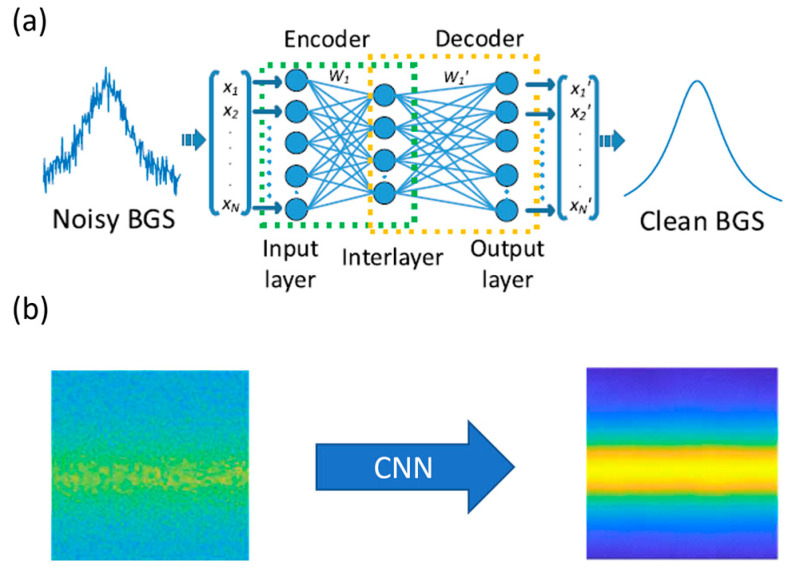
(**a**) Single Brillouin gain spectrum denoising using autoencoder-based neural networks. Reprinted with permission from [124] © IEEE; (**b**) 2D Brillouin gain spectrum denoising using convolutional neural networks. Adapted with permission from [125] © IEICE.

Several neural network architectures have been proposed. Wang B. et al. [123,124] reported on BGS denoising using an encoder/decoder structure, as shown in Figure 10a. This structure consists of an input layer, an intermediate layer and an output layer. The input corresponds to the noisy BGS, while the output to the clean BGS. Therefore, the network learns to map the noisy BGS to the clean (denoised) BGS. The intermediate layer was used for dimension reduction and feature extraction. Furthermore, once the training of the model was finished, the authors used the outputs of the intermediate layer to directly predict temperature without applying LCF. In other words, they built a stacked neural network architecture combining the encoder network with the previously described ANNs for temperature extraction. The training of the encoder/decoder neural network was performed using synthetic data, which consisted of additive Gaussian white noise. The stacked neural network was tested with BOTDA experimental data.

Wu et al. [126] and Zheng et al. [127] proposed CNNs that consider the spatial and spatio-temporal similarities, respectively. Specifically, the CNNs demonstrated by Wu et al. [126] accept 2D BGSs (Figure 10b) with the dimensions defined by the number of frequency scanning points and the number of the spatially resolved sensing points. They reported that the BM3D had a negative effect on the system’s set spatial resolution, which was not observed when CNN denoisers were used. Zheng et al. [127] designed a CNN with three dimensions including the time. The authors concluded that the 3D CNN provides higher SNR than the 2D CNN, with the reported improvement being 3.6 dB. However, we note that the SNR improvement is expected to be related e.g., to the number of signal averages during the experiments.

The results of these two papers indicated that the CNN denoisers are approximately more than two orders of magnitude faster than the conventional BM3D denoiser. This enabled the real-time denoising of the experimentally obtained BGS allowing for even dynamic strain sensing [127]. However, we need to mention that even though the denoising itself is fast, the training of the CNNs is time-consuming, and it can last up to 45 h as reported in [126]. It is of high importance to note that these training times were acquired using a state-of-the-art GPU. The use of a CPU is expected to increase the training time dramatically.

Very recently, Yang et al. [125] proposed a 2D CNN, namely attention-guided denoising CNN, which has been widely used in the field of image recognition to shorten the computation time of deep CNN architectures [128,129]. The authors claimed that the new CNN architecture could result in more accurate BFS estimations than the one used in [126]. However, more investigations including experimental data are required.

Even though neural network-based denoisers resulted in BGSs with high SNR and short computation times, more investigations are required for a wider use in the future. As mentioned previously, no optimization is performed once the denoising model is trained, which renders the CNN denoisers faster than the BM3D conventional image denoising method, as reported in [126]. To the best of our knowledge, a similar comparison between CNN denoisers and other conventional denoising algorithms, such as non-local means (NLM) and wavelet denoising (WD) using GPUs, has not been reported yet. Nevertheless, a comparison between the three denoising algorithms, BM3D, NLM and WD, using a CPU showed that WD is two orders of magnitude faster than the BM3D and NLM [130]. Therefore, even if the CNN denoisers are faster than the BM3D, further studies should investigate whether the CNN denoisers are faster than the WD as well. We note that the use of the same hardware (i.e., GPU) is of high importance when computation times are compared.

A limitation of the neural network denoisers that needs to be addressed in the future arises from the fact that the size of the input images should always match the network’s input size. This means that all the images should consist of the same number of sampling points and the same number of frequencies. For this reason, methods to address this issue, such as zero-padding and interpolation, should be tested [131].

### 3.3. Machine Learning for Temperature and Strain Predictions Directly from the Brillouin Gain Spectrum

Machine learning has also been used to extract temperature directly from BGSs. Azad et al. [132,133] and Wang L. et al. [134] proposed a signal post-processing method based on ANNs to predict temperature without extracting the BFS. First, an ANN was trained based on the normalized BGS corresponding to different temperatures. The training dataset consisted of ideal synthetic data with varying linewidths. We note that in contrast to other training datasets, Azad et al. [133] did not add noise to the ideal synthetic data.

**Figure 11:**
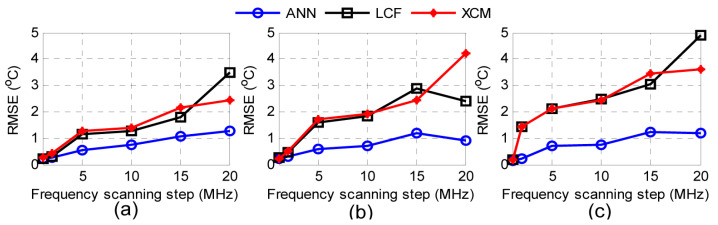
Performance comparison of the ANN, LCF and XCM in terms of the temperature RMSE when the fiber is heated to 29.90 °C (**a**), 39.14 °C (**b**) and 48.63 °C (**c**). Reprinted with permission from [133] © Optica Publishing Group.

The authors trained separate ANNs for BGSs recorded using different frequency scanning steps. This results from the fact that the set frequency scanning step affects the number of data points of the BGS, and thus, ANNs with different nodes in the input layer are required. Figure 11 compares the performance of the ANN to that of the LCF and cross-correlation method (XCM) when different frequency scanning steps are used. The performance is calculated in terms of the temperature RMSE when the fiber is exposed to controlled-temperature conditions. In general, the ANNs perform better than the conventional methods, which according to the authors, is attributed to the fact that the ANNs are trained and optimized for each frequency step separately. However, we observe that the ANNs perform significantly better than the conventional methods when the set frequency step is greater than 2. These results agree with those reported by Wang J. et al. [135] and Cao et al. [136] and indicate that ANNs can handle sparse data very well.

Madaschi et al. [137] proposed a similar ANN for direct temperature extraction that could handle BGS acquired with different frequency scanning steps. Specifically, they applied spline interpolation based on the BGS, so that the data points of the BGS are equal to the number of nodes in the input layer of the ANN. This solution increases the flexibility of the ANN, but according to the authors, the extracted temperature accuracy of this approach is slightly lower than the temperature accuracy of the separately trained ANNs. We note that a BGS interpolation has also been proposed and tested by Liang et al. [92] for BFS extraction as mentioned in the previous chapter.

Azad et al. [133] and Madaschi et al. [137] highlighted the improvement in terms of computation time that the ANNs offer in comparison to the conventional methods. Both reports agree that the temperature extraction through ANNs can be even two orders of magnitude faster than the LCF approach.

Li et al. [138] studied the impact of the training dataset on the temperature accuracy of the ANNs. Specifically, they created three different training datasets using synthetic BGS consisting of (a) Lorentzian functions, (b) Pseudo-Voigt functions and (c) Pseudo-Voigt functions with artificial noise. The authors tested the three different trained models on data collected by a BOTDR system and concluded that the model trained with noisy Pseudo-Voigt functions delivered the most accurate temperature predictions. However, because the shape of the BGS that is obtained by systems that are based on pump pulses, such as BOTDR and BOTDA, depends on the pump pulse power and width [88,139], a general conclusion cannot be drawn.

The implementation of ANNs for temperature extraction has been also studied by other research groups [123,140,141,142,143,144]. For example, Wang M. et al. [141] brought together the state-of-the-art ANN-based signal processing with the internet of things (IoT) [145] to facilitate automatization and enhance data management and analytics.

Zhang et al. [146] extracted temperature, applying kernel extreme learning machines (K-ELM). ELM is a special case of ANNs consisting of a single hidden layer, where the first weight matrix is randomly initialized [147,148]. This means that only the last weight matrix is optimized, and thus, the training is faster. K-ELM is a modified version of the simple ELM that introduces intrinsic kernel mapping [147]. In comparison to the simple ELM, the K-ELM algorithm does not require either the number of nodes in the hidden layer to be specified or the feature mapping to be known. According to Zhang et al. [146], K-ELM proved to be very robust and in comparison to the conventional LCF approach, they slightly reduced the extracted temperature error by 0.3 °C and improved the temperature extraction time by 120 times. The authors also applied simple ELM and found that they perform significantly worse than the conventional LCF.

Apart from neural networks, SVMs have also been applied to extract temperature from BGSs [149]. SVMs are simpler than ANNs, and fewer hyperparameters need to be optimized. Furthermore, the SVM average training procedure is significantly faster than that of the ANNs. Wu et al. [149] used SVMs to extract temperature and concluded that SVMs outperform the conventional LCF when the SNR of the data is low. At high SNR values, the temperature accuracy of the SVM is comparable with that of the LCF method. The authors stated that these results are very promising for long-range sensing because, at distant positions, the SNR is significantly lower. Furthermore, the performance difference between the SVMs and the LCF increases with the frequency scanning step. This agrees completely with the results shown in [133] and indicates that not only the ANNs but also the SVMs can handle sparse data very well. Wu et al. [149] also mentioned that the training time, as well as the prediction time, is very short. As an example, the training time of an SVM was approximately 1 s, while the prediction time of 101,500 BGSs was less than 16 s. We note that even though the prediction times of the SVMs and the ANNs are similar, the SVMs can be trained much faster than the ANNs. In another paper, the same authors used PCA to further reduce the data processing time without sacrificing temperature accuracy [150]. The results reported by the authors indicate that the PCA reduced the prediction time by up to 20%.

Nordin et al. [151,152,153] proposed the use of GLM to extract temperature. GLM is a generalized form of linear regression that does not assume that the response variables (targets) are normally distributed. Similar to the previously mentioned machine learning algorithms, GLM is capable of predicting the temperature directly from the BGS without estimating the BFS. The authors concluded that GLM extracts temperature faster and more accurately than the conventional LCF. Specifically, the temperature extraction time was approximately two orders of magnitude faster than the LCF, while the temperature error improvement varied from approximately 0.4 °C to 5 °C, depending on the frequency-tuning step and the temperature conditions. The authors in [151] concluded that GLM in combination with conventional BFS extraction methods, such as LCF, results in a significant increase in temperature accuracy even when the SNR is low. The most important characteristic of the GLM is the easy interpretation, which arises from the algorithm’s simplicity and its straightforward implementation.

In another publication, Nordin et al. [154] trained different machine learning algorithms for direct temperature extraction and found that random forest performs slightly better than the GLM in terms of temperature precision. We note that random forest is an ensemble of decision trees that usually outperforms single decision trees but at the cost of complexity [155]. The authors also applied ANNs, but surprisingly, they found that they perform worse than the conventional LCF. This is in contrast to all the aforementioned studies [132,133,134,141] that showed that ANNs outperform the conventional LCF. However, we note that in comparison to other machine learning algorithms, such as random forest, SVM and GLM, ANNs require, in general, much larger datasets and the hyperparameter tuning is more complex and time-consuming. Therefore, the relatively low ANN performance reported by Nordin et al. [154] may be attributed to an insufficient dataset or to a not well-optimized neural network structure.

Apart from direct temperature extraction, similar machine learning approaches have been proposed for direct strain extraction [156,157,158,159,160]. As an example, we mention that Song et al. in 2020 proposed deep ANNs to detect microcracks in structural elements [156]. Even though the algorithm performed very well, in 2021 they made use of PCA and SVM for the same purpose, asserting that the deep ANNs were difficult to implement and interpret [158].

In comparison to the approaches presented in Section 3.1, the temperature or strain predictions directly from the BGS represents a more compact solution and allows for predictions based not only on the BFS but also on other features that can be extracted from the BGS, such as linewidth and gain. Because, in many cases, these features depend on the experimental settings, e.g., pulse width and power, most of the authors trained the machine learning models using synthetic data, so that no relationship between linewidth or gain and the measurand can be learned. However, we note that the use of additional features can potentially result in improved temperature errors, and this can be investigated in the future.

Due to temperature and strain cross-sensitivity, the direct temperature (or strain) extraction from the BGS can completely fail if strain (or temperature) changes occur. This is a clear disadvantage compared to the previous approaches described in the Section 3.1, and thus, methods to extract temperature and strain simultaneously using machine learning have also been proposed.

Researchers used machine learning to simultaneously predict two parameters addressing the well-known cross-sensitivity problem. This is of great importance for accurate temperature or strain monitoring but also for industrial applications, where simultaneous temperature and strain monitoring is needed.

**Figure 12:**
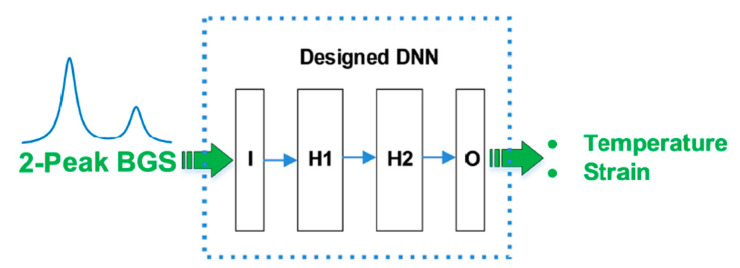
Simultaneous temperature and strain extraction using a two-peak Brillouin gain spectrum from a large effective area fiber (LEAF) and artificial neural networks. I: input layer; H1: first hidden layer; H2: second hidden layer; O: output layer. Reprinted with permission from [72] © Optica Publishing Group.

Wang B. et al. [72] proposed ANNs for temperature and strain discrimination using a LEAF fiber. LEAF fibers are characterized by a BGS with two peaks, as illustrated in Figure 12. These two peaks have different temperature and strain sensitivities, which means that the two parameters could be decoupled even with the conventional equation-solving method as described in Section 2. However, if the SNR is low, the conventional approach comes at the cost of large errors, which does not allow for any practical application. Wang B. et al. [72] trained the ANN with a synthetic double-peak BGS. The ANN was tested not only on synthetic data but also on BOTDA experimental data resulting from an optical fiber of 24 km. They concluded that ANNs provide temperature and strain RMSE of 4.2 °C and 134.2 με, respectively. These temperature and strain errors were approximately seven and five times lower than those obtained from the conventional equation-solving method, respectively.

Yang et al. [73] followed a similar methodology but used one-dimensional CNNs instead of ANNs. Specifically, they used a synthetic two-peak BGS and experimental data to train and test the CNN, respectively. They employed an approximately 20 km optical fiber and concluded that CNNs provide a temperature and strain RMSE of 2 °C and 32.3 με, respectively.

Ruiz-Lombera et al. [71] reported on simultaneous temperature and strain sensing in a standard optical fiber using PCA and ANN, but using a classification instead of a regression algorithm. Specifically, the ANN was trained to predict 40 temperature and strain classes, in total. The temperature and strain ranges were from 22 °C to 62 °C and from 0 με to 1536 με, respectively. With the hyperparameters of the ANN being optimized, the classification rate reached almost 90%. Even though the classification accuracy is high, we have to note that the set temperature and strain steps were 10 °C and approximately 200 με, respectively.

The majority of the authors estimated the performance of their machine learning models in terms of the BFS, temperature or strain error. However, we need to note that the reported performances do not depend only on the applied machine learning algorithm, but on a plethora of factors, such as the experimental parameters (length of the fiber, spatial resolution, measurement settings [161]), the error estimation methodology and metric, the stability of the climate chambers, the accuracy of the reference sensors, the precision of the fiber optic stretchers, etc.

Apart from accuracy, many authors estimated the performance of their methods by considering the prediction time. However, this criterion alone cannot be used to compare the various reported machine learning methodologies. This limitation arises from the fact that prediction time is influenced not only by the machine learning algorithm itself but also by the hardware and software utilized. Factors, such as the type and number of CPU threads, the computational power of the GPU and the machine learning framework employed (e.g., Keras, PyTorch, TensorFlow), strongly affect the prediction time [92,100,162]. Consequently, it is not reliable to compare previously employed methodologies solely based on errors or the prediction time. Hence, it is crucial to carefully consider the context and specific details of each study when evaluating the reported performance of machine learning algorithms.

To enhance the understanding of the appropriate application and suitability of each algorithm, a comprehensive table is provided below (Table 1), outlining the strengths and weaknesses of the employed machine learning methodologies.

## 4. Machine Learning Applied in Brillouin Frequency Domain Sensors

This section discusses the advances in machine learning-assisted BOFDA sensors, in particular. In contrast to BOTDA, where the pulse response is measured directly, in BOFDA, the pulse response is retrieved by applying inverse fast Fourier transformations to the obtained complex transfer function, as described in Section 2. This has the advantage that no ultra-fast electronics are required, which, on the one hand, has a positive impact on the system’s cost but, on the other hand, increases the measurement time significantly. For this reason, a machine learning method for time-efficient BOFDA measurements was proposed [69,167].

**Figure 13:**
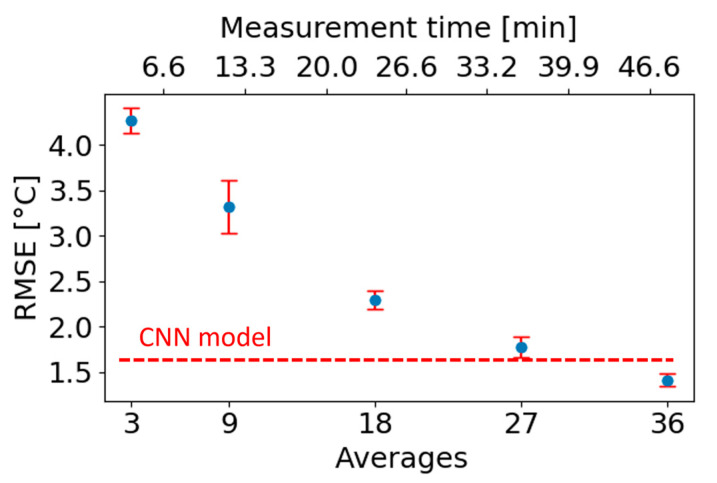
Temperature RMSE of the conventional LCF method (blue dots) vs. the measurement time. The dashed red line corresponds to the CNN performance based on data obtained using 4 min measurements. Adapted from [69]. Copyright 2021 licensed under a Creative Commons Attribution 4.0 International license.

BOFDA measurements can be shortened by reducing the number of averages, but this comes at the cost of a lower SNR. Figure 13 shows the relationship between temperature error resulting from the conventional LCF method and measurement time (or the number of averages). The dashed red line corresponds to the CNN performance based on low SNR data obtained using 4 min measurements. The results show that the LCF reaches the performance of the CNN model after 36 min, which indicates that the application of the CNN resulted in a nine-fold measurement time reduction. We note that these results agree with other studies that showed that CNNs are tolerant to noise [68,99]. Furthermore, the long measurement time is a drawback of BOFDA when compared to BOTDA, and thus, the time reduction is of great importance for its wider application in the future.

**Figure 14:**
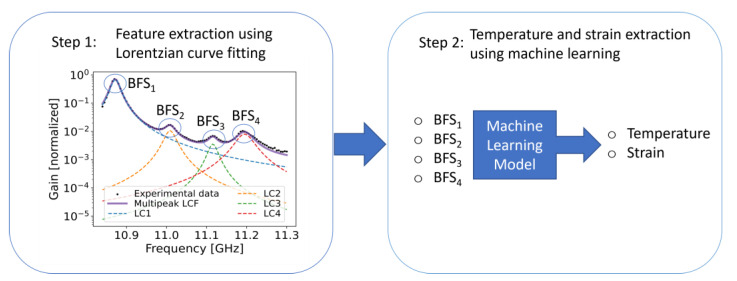
Temperature and strain discrimination using the BFS extracted via conventional Lorentzian curve-fitting. Adapted with permission from [70] © Optica Publishing Group.

Besides the measurement time, the problem of cross-sensitivity is also of great importance towards a wider use of BOFDA in industrial applications in the future, and thus, Karapanagiotis et al. [70] proposed simple machine learning to discriminate temperature and strain in standard telecom optical fibers. The use of these fibers opens the way for fiber optic monitoring using the already existing laid-out fiber optic networks. The authors demonstrated a BOFDA system of high SNR to obtain the multipeak spectrum of the legacy standard SMF28^®^ (Corning^®^) optical fiber. The multipeak spectrum of the standard fiber is not easily obtainable, and thus, a high SNR is required [168]. That spectrum is characterized by three secondary peaks, of which the amplitude is more than two orders of magnitude lower than the fundamental. The BFSs were extracted using the conventional LCF method, as described in the Section 2. Ridge regression [155] ,which is nothing more than a simple polynomial regression, including a penalty term to avoid overfitting, was used. The algorithm managed to capture nonlinearities in the data and delivered temperature and strain errors of 2.6 °C and 58 με, respectively. We note that both the training and test datasets consisted of experimental data, and the errors were calculated using cross-validation. Gaussian process regression (GPR) [169], which is based on Bayesian statistics was also used to extract temperature and strain and delivered 22% lower temperature and strain errors than the ridge regression. We note that the optical fiber’s total length was approximately 400 m, and the temperature and strain errors resulting from the equation-solving method were 5 °C and 114 με, respectively. The proposed methodology is shown in Figure 14.

Apart from temperature and strain discrimination, temperature and relative humidity effects were also decoupled by using a humidity sensitive Polyimide (PI)-coated optical fiber [170,171]. We note that humidity causes the PI coating to sweal, which in turn, induces strain to the optical fiber, and thus, the BFS changes. Due to the high SNR of the system, the authors managed again to obtain a multipeak spectrum and followed a similar methodology with [70]. The difference lies in the fact that the temperature and humidity effects could not be separated by using only the BFS, and thus, the linewidths were also employed. Algorithms, such as ridge regression, decision trees and ANNs, were used. ANNs seemed to outperform the other algorithms delivering temperature and relative humidity errors of 0.9 °C and 6.5%RH, respectively.

Unlike the previous machine learning approaches for temperature and strain discrimination in BOTDA sensors [71,72,73], which used the entire BGS as input, these last papers employed, as inputs, spectral parameters extracted via LCF. The advantage of extracting features is that they render the interpretability easier. For example, in [171] the authors used backward feature elimination [155] to study the feature importance and found that only the features extracted from the first two peaks contributed to the algorithm’s decision. This finding indicates that half of the spectrum does not need to be obtained, which positively affects the measurement time. However, we need to mention that the feature extraction via LCF may be challenging in cases of low SNR.

We note that these methods, as described above, can potentially be combined so that simultaneous multiparameter sensing, including temperature, strain and humidity, is demonstrated. Specifically, this could be achieved by applying machine learning and using the two-fiber configuration, including an acrylate-coated fiber and a PI-coated fiber, placed in parallel and close to the other. With the acrylate-coated fiber measuring strain and temperature and the PI-coated fiber measuring humidity, temperature and strain, a multiparameter Brillouin DFOS could be feasible.

The aforementioned algorithms can also be employed in other Brillouin DFOS systems (e.g., BOTDA and BOCDA) providing that those sensors are able to record a high SNR multipeak BGS similar to the one shown in Figure 14. This results from the fact, that the authors in [70,170,171] made use of spectral properties that can be extracted via conventional LCF in all Brillouin DFOS systems.

Time domain systems are more commonly employed in both research and industry compared to frequency domain systems. As a result, the majority of machine learning approaches have been primarily implemented in the context of time domain systems. Nevertheless, it is worth noting that in many instances, machine learning methodologies employed in time domain systems can be readily adapted and applied to frequency domain systems as well.

## 5. Conclusions and Future Perspectives

We reviewed machine learning approaches applied in Brillouin DFOSs. In recent years, Brillouin DFOSs have been greatly impacted by the emergence of machine learning. This is attributed to the opportunities for advanced signal processing that the sensing data provide, the already reported successful applications of machine learning in other fields of research and the advancements in computational capabilities with GPU. Moreover, machine learning offers solutions that do not significantly increase the cost of the system, except for a small increment in the case of using a GPU.

Machine learning algorithms have been employed to the signal processing of the Brillouin DFOS to extract features (such as BFSs and linewidths) or directly predict temperature, strain or humidity from the BGS. Due to the problem of cross-sensitivity in optical fibers, machine learning approaches that output one parameter (e.g., temperature) are prone to errors, if the other parameters (e.g., strain or humidity) are altered. To this end, machine learning approaches for decoupling two parameters even in standard optical fibers, have been reported. Furthermore, machine learning has been applied for denoising, and reports showed that they can potentially outperform well-known denoising algorithms, such as BM3D and NLM.

While most of the machine learning approaches can be applied to Brillouin DFOS systems, regardless of whether they operate in the time or frequency domain, there are some approaches that have been specifically tailored to specific systems. For instance, machine learning has enabled a simple BOTDA system to achieve the same spatial resolution as a more complex DPP-BOTDA setup. Additionally, in BOFDA sensors, machine learning contributed to a significant reduction of the measurement time, which is expected to render BOFDA more attractive for applications in the field.

In the future, machine learning can also be combined with other newly developed signal processing techniques. Recently, compressed sensing, for example, has gained increasing attention for reconstructing signals that have been sampled below the Nyquist frequency [172]. Compressed sensing has already been applied in Brillouin DFOSs to reduce the recorded data and consequently, to shorten the measurement time [173,174,175]. We believe that compressed sensing in combination with machine learning will contribute to the further development of Brillouin DFOSs. We note that the combination of machine learning and compressed sensing is already known in the literature as compressed learning [176].

In this paper, we highlighted the achievements that machine learning has brought in Brillouin DFOSs, and we also clarified the weaknesses, so that the limits will be pushed even further in the future. Among the most important weaknesses of the proposed methodologies is related to the interpretability. However, we believe that with the help of new techniques that recently shed light on complex machine learning algorithms, we will soon start witnessing an increasing number of interpretable machine learning-assisted Brillouin DFOS systems. The interpretation of the models will render the hyperparameter optimization process more efficient and will facilitate the release of industrial machine learning systems. We hope that this review will contribute towards further investigations in the future.

## Figures and Tables

**Table 1:** Comparison of the strengths and weaknesses of the applied machine learning algorithms. The last column provides the references that apply the corresponding machine learning algorithms.

Algorithm	Strengths	Weaknesses	References
AΝΝ/CNN	Handle complex patterns and relationships in large data	Time-consuming training, requires a large amount of data	[68,92,97,98,99,101,102,103,116,123,124,125,126,127,132,133,134,135,136,137,138,141,146,156,160,161,163,164]
KNN	No training is required, simple and intuitive	Relatively slow predictions	[114,115]
SVM	Fast training and predictions, works well with small datasets	Not suitable for large datasets	[107,149,150,158,165,166]
GLM	Easy to interpret	Difficult to handle non-linear and complex data	[144,151,153,154]

## Data Availability

Not applicable.

## Abbreviations

The following abbreviations are used in this manuscript:

ANN	artificial neural network
BOTDA	Brillouin time domain analysis
BOTDR	Brillouin time domain reflectometry
BOFDA	Brillouin frequency domain analysis
BOFDR	Brillouin frequency domain reflectometry
BOCDA	Brillouin correlation domain analysis
BOCDR	Brillouin correlation domain reflectometry
BFS	Brillouin frequency shift
BGS	Brillouin gain spectrum
BM3D	block-matching and 3D filtering
CNN	convolutional neural network
CPU	central processing unit
DOFS	distributed fiber optic sensors
ELM	extreme learning machine
GLM	generalized linear model
GPR	Gaussian process regression
GPU	graphics processing unit
IFFT	inverse fast Fourier transformation
IoT	internet of things
K-ELM	kernel extreme learning machine
KNN	k-nearest neighbors
LCF	Lorentzian curve fitting
LEAF	large effective area fiber
MSE	mean square error
NLM	non-local means
PCA	principal component analysis
RF	random forest
RMSE	root mean square error
SNR	signal-to-noise ratio
SVM	support vector machines
WD	wavelet denoising
XCM	cross-correlation method

## References

[1] Ecke W., Nöther N., Peters K.J., Wosniok A., Krebber K., Meyendorf N.G., Thiele E. A distributed fiber optic sensor system for dike monitoring using Brillouin optical frequency domain analysis. Proceedings of the Smart Sensor Phenomena, Technology, Networks, and Systems.

[2] Schenato L. (2017). A Review of Distributed Fibre Optic Sensors for Geo-Hydrological Applications. Appl. Sci..

[3] Bado M.F., Casas J.R. (2021). A Review of Recent Distributed Optical Fiber Sensors Applications for Civil Engineering Structural Health Monitoring. Sensors.

[4] Monsberger C.M., Bauer P., Buchmayer F., Lienhart W. (2022). Large-scale distributed fiber optic sensing network for short and long-term integrity monitoring of tunnel linings. J. Civ. Struct. Health.

[5] Wu T., Liu G., Fu S., Xing F. (2020). Recent Progress of Fiber-Optic Sensors for the Structural Health Monitoring of Civil Infrastructure. Sensors.

[6] Stajanca P., Chruscicki S., Homann T., Seifert S., Schmidt D., Habib A. (2018). Detection of Leak-Induced Pipeline Vibrations Using Fiber—Optic Distributed Acoustic Sensing. Sensors.

[7] Matsumoto H., Araki E., Kimura T., Fujie G., Shiraishi K., Tonegawa T., Obana K., Arai R., Kaiho Y., Nakamura Y. (2021). Detection of hydroacoustic signals on a fiber-optic submarine cable. Sci. Rep..

[8] Fang G., Li Y.E., Zhao Y., Martin E.R. (2020). Urban Near-Surface Seismic Monitoring Using Distributed Acoustic Sensing. Geophys. Res. Lett..

[9] Sladen A., Rivet D., Ampuero J.P., De Barros L., Hello Y., Calbris G., Lamare P. (2019). Distributed sensing of earthquakes and ocean-solid Earth interactions on seafloor telecom cables. Nat. Commun..

[10] Spica Z.J., Perton M., Martin E.R., Beroza G.C., Biondi B. (2020). Urban Seismic Site Characterization by Fiber-Optic Seismology. J. Geophys. Res. Solid Earth.

[11] Masoudi A., Pilgrim J.A., Newson T.P., Brambilla G. (2019). Subsea Cable Condition Monitoring with Distributed Optical Fiber Vibration Sensor. J. Light. Technol..

[12] Min R., Liu Z., Pereira L., Yang C., Sui Q., Marques C. (2021). Optical fiber sensing for marine environment and marine structural health monitoring: A review. Opt. Laser Technol..

[13] Thomas P.J., Hellevang J.O. (2017). A fully distributed fibre optic sensor for relative humidity measurements. Sens. Actuators B Chem..

[14] Stajanca P., Hicke K., Krebber K. (2019). Distributed Fiberoptic Sensor for Simultaneous Humidity and Temperature Monitoring Based on Polyimide-Coated Optical Fibers. Sensors.

[15] He C., Korposh S., Correia R., Liu L., Hayes-Gill B.R., Morgan S.P. (2021). Optical fibre sensor for simultaneous temperature and relative humidity measurement: Towards absolute humidity evaluation. Sens. Actuators B Chem..

[16] Schenato L., Galtarossa A., Pasuto A., Palmieri L. (2020). Distributed optical fiber pressure sensors. Opt. Fiber Technol..

[17] Jaroszewicz L.R., Kusche N., Schukar V., Hofmann D., Basedau F., Habel W., Woschitz H., Lienhart W. Field examples for optical fibre sensor condition diagnostics based on distributed fibre optic strain sensing. Proceedings of the 5th European Workshop on Optical Fibre Sensors.

[18] Stajanca P., Mihai L., Sporea D., Neguţ D., Sturm H., Schukar M., Krebber K. (2016). Effects of gamma radiation on perfluorinated polymer optical fibers. Opt. Mater..

[19] Stajanca P., Krebber K. (2017). Radiation-Induced Attenuation of Perfluorinated Polymer Optical Fibers for Radiation Monitoring. Sensors.

[20] Lewis E., Wosniok A., Sporea D., Neguţ D., Krebber K. Gamma radiation influence on silica optical fibers measured by optical backscatter reflectometry and Brillouin sensing technique. Proceedings of the 6th European Workshop on Optical Fibre Sensors.

[21] Rizzolo S., Boukenter A., Ouerdane Y., Michalon J.-Y., Marin E., Macé J.-R., Girard S. (2020). Distributed and discrete hydrogen monitoring through optical fiber sensors based on optical frequency domain reflectometry. J. Phys. Photonics.

[22] Lin Y., Liu F., He X., Jin W., Zhang M., Yang F., Ho H.L., Tan Y., Gu L. (2017). Distributed gas sensing with optical fibre photothermal interferometry. Opt. Express.

[23] Hartog A.H. (2017). An Introduction to Distributed Optical Fibre Sensors.

[24] Taranov M.A., Gorshkov B.G., Alekseev A.E. (2020). Achievement of an 85 km Distance Range of Strain (Temperature) Measurements Using Low-Coherence Rayleigh Reflectometry. Instrum. Exp. Tech..

[25] Lu Z., Feng T., Li F., Yao X.S. (2023). Optical Frequency-Domain Reflectometry Based Distributed Temperature Sensing Using Rayleigh Backscattering Enhanced Fiber. Sensors.

[26] Pedraza A., del Río D., Bautista-Juzgado V., Fernández-López A., Sanz-Andrés Á. (2023). Study of the Feasibility of Decoupling Temperature and Strain from a ϕ-PA-OFDR over an SMF Using Neural Networks. Sensors.

[27] Palmieri L., Schenato L., Santagiustina M., Galtarossa A. (2022). Rayleigh-Based Distributed Optical Fiber Sensing. Sensors.

[28] Bernini R., Minardo A., Zeni L. (2009). Dynamic strain measurement in optical fibers by stimulated Brillouin scattering. Opt. Lett..

[29] Voskoboinik A., Yilmaz O.F., Willner A.W., Tur M. (2011). Sweep-free distributed Brillouin time-domain analyzer (SF-BOTDA). Opt. Express.

[30] Zhou D., Dong Y., Wang B., Pang C., Ba D., Zhang H., Lu Z., Li H., Bao X. (2018). Single-shot BOTDA based on an optical chirp chain probe wave for distributed ultrafast measurement. Light Sci. Appl..

[31] Minardo A., Porcaro G., Giannetta D., Bernini R., Zeni L. (2013). Real-time monitoring of railway traffic using slope-assisted Brillouin distributed sensors. Appl. Opt..

[32] Motil A., Bergman A., Tur M. (2016). [INVITED] State of the art of Brillouin fiber-optic distributed sensing. Opt. Laser Technol..

[33] Sun X., Yang Z., Hong X., Jin S., Luo J., Soto M.A., Wu J. (2022). Ultra-long Brillouin optical time-domain analyzer based on distortion compensating pulse and hybrid lumped–distributed amplification. APL Photonics.

[34] Zhang L., Wang Z., Li J., Zeng J., Li Y., Jia X., Rao Y. (2015). Ultra-long dual-sideband BOTDA with balanced detection. Opt. Laser Technol..

[35] Soto M.A., Bolognini G., Di Pasquale F. (2011). Optimization of long-range BOTDA sensors with high resolution using first-order bi-directional Raman amplification. Opt. Express.

[36] Denisov A., Soto M.A., Thévenaz L. (2016). Going beyond 1000000 resolved points in a Brillouin distributed fiber sensor: Theoretical analysis and experimental demonstration. Light Sci. Appl..

[37] Bernini R., Minardo A., Zeni L. (2012). Distributed Sensing at Centimeter-Scale Spatial Resolution by BOFDA: Measurements and Signal Processing. IEEE Photonics J..

[38] Sperber T., Eyal A., Tur M., Thévenaz L. (2010). High spatial resolution distributed sensing in optical fibers by Brillouin gain-profile tracing. Opt. Express.

[39] Garus D., Gogolla T., Krebber K., Schliep F. (1996). Distributed sensing technique based on Brillouin optical-fiber frequency-domain analysis. Opt. Lett..

[40] Jayawickrema U.M.N., Herath H.M.C.M., Hettiarachchi N.K., Sooriyaarachchi H.P., Epaarachchi J.A. (2022). Fibre-optic sensor and deep learning-based structural health monitoring systems for civil structures: A review. Measurement.

[41] Kandamali D.F., Cao X., Tian M., Jin Z., Dong H., Yu K. (2022). Machine learning methods for identification and classification of events in ϕ-OTDR systems: A review. Appl. Opt..

[42] Shiloh L., Eyal A., Giryes R. (2019). Efficient Processing of Distributed Acoustic Sensing Data Using a Deep Learning Approach. J. Light. Technol..

[43] Ohodnicki P.R., Zhang P., Lalam N., Karki D., Venketeswaran A., Babaee H., Wright R. Fusion of Distributed Fiber Optic Sensing, Acoustic NDE, and Artificial Intelligence for Infrastructure Monitoring. Proceedings of the 27th International Conference on Optical Fiber Sensors.

[44] Shiloh L., Eyal A., Giryes R. Deep Learning Approach for Processing Fiber-Optic DAS Seismic Data. Proceedings of the 26th International Conference on Optical Fiber Sensors.

[45] Shi Y., Wang Y., Wang L., Zhao L., Fan Z. (2020). Multi-event classification for Φ-OTDR distributed optical fiber sensing system using deep learning and support vector machine. Optik.

[46] Shi Y., Wang Y., Zhao L., Fan Z. (2019). An Event Recognition Method for Φ-OTDR Sensing System Based on Deep Learning. Sensors.

[47] Peng Z., Jian J., Wen H., Gribok A., Wang M., Liu H., Huang S., Mao Z.-H., Chen K.P. (2020). Distributed fiber sensor and machine learning data analytics for pipeline protection against extrinsic intrusions and intrinsic corrosions. Opt. Express.

[48] Li S., Peng R., Liu Z. (2020). A surveillance system for urban buried pipeline subject to third-party threats based on fiber optic sensing and convolutional neural network. Struct. Health Monit..

[49] Bai Y., Xing J., Xie F., Liu S., Li J. (2019). Detection and identification of external intrusion signals from 33 km optical fiber sensing system based on deep learning. Opt. Fiber Technol..

[50] Chen J., Wu H., Liu X., Xiao Y., Wang M., Yang M., Rao Y. A Real-Time Distributed Deep Learning Approach for Intelligent Event Recognition in Long Distance Pipeline Monitoring with DOFS. Proceedings of the 2018 International Conference on Cyber-Enabled Distributed Computing and Knowledge Discovery (CyberC).

[51] Wu Z., Wang Q., Gribok A.V., Chen K.P. Pipeline Degradation Evaluation Based on Distributed Fiber Sensors and Convolutional Neural Networks (CNNs). Proceedings of the 27th International Conference on Optical Fiber Sensors.

[52] Wang Q., Jian J., Wang M., Wu J., Mao Z.-H., Gribok A.V., Chen K.P. (2020). Pipeline Defects Detection and Classification Based on Distributed Fiber Sensors and Neural Networks. Optical Fiber Sensors Conference 2020 Special Edition.

[53] Wu H., Chen J., Liu X., Xiao Y., Wang M., Zheng Y., Rao Y. (2019). One-Dimensional CNN-Based Intelligent Recognition of Vibrations in Pipeline Monitoring with DAS. J. Light. Technol..

[54] Li Z., Zhang J., Wang M., Zhong Y., Peng F. (2020). Fiber distributed acoustic sensing using convolutional long short-term memory network: A field test on high-speed railway intrusion detection. Opt. Express.

[55] Wang Z., Zheng H., Li L., Liang J., Wang X., Lu B., Ye Q., Qu R., Cai H. (2019). Practical multi-class event classification approach for distributed vibration sensing using deep dual path network. Opt. Express.

[56] Kowarik S., Hussels M.-T., Chruscicki S., Münzenberger S., Lämmerhirt A., Pohl P., Schubert M. (2020). Fiber Optic Train Monitoring with Distributed Acoustic Sensing: Conventional and Neural Network Data Analysis. Sensors.

[57] Hamadi A., Montarsolo E., Kabalan A., Garbini G.P., Hammi T. (2020). Machine Learning Based Analysis of Optical Fiber Sensing Intensity Data for Train Tracking Application. Optical Fiber Sensors Conference 2020 Special Edition.

[58] Hernandez P.D., Ramirez J.A., Soto M.A. (2022). Deep-Learning-Based Earthquake Detection for Fiber-Optic Distributed Acoustic Sensing. J. Light. Technol..

[59] van den Ende M., Lior I., Ampuero J.-P., Sladen A., Ferrari A., Richard C. (2021). A Self-Supervised Deep Learning Approach for Blind Denoising and Waveform Coherence Enhancement in Distributed Acoustic Sensing Data. IEEE Trans. Neural Netw. Learn. Syst..

[60] Wang M., Deng L., Zhong Y., Zhang J., Peng F. (2021). Rapid Response DAS Denoising Method Based on Deep Learning. J. Light. Technol..

[61] Zhong T., Cheng M., Lu S., Dong X., Li Y. (2022). RCEN: A Deep-Learning-Based Background Noise Suppression Method for DAS-VSP Records. IEEE Geosci. Remote Sens. Lett..

[62] Yang L., Fomel S., Wang S., Chen X., Chen W., Saad O.M., Chen Y. (2022). Denoising of distributed acoustic sensing data using supervised deep learning. Geophysics.

[63] Liehr S., Borchardt C., Münzenberger S. (2020). Long-distance fiber optic vibration sensing using convolutional neural networks as real-time denoisers. Opt. Express.

[64] Wang Y., Liu Q., Li B., Chen D., Li H., He Z. (2020). Boosting the data processing speed by artificial neural network in distributed fiber-optic sensor. Optical Fiber Sensors Conference 2020 Special Edition.

[65] Liehr S., Jäger L.A., Karapanagiotis C., Münzenberger S., Kowarik S. (2019). Real-time dynamic strain sensing in optical fibers using artificial neural networks. Opt. Express.

[66] Venketeswaran A., Lalam N., Wuenschell J., Ohodnicki P.R., Badar M., Chen K.P., Lu P., Duan Y., Chorpening B., Buric M. (2021). Recent Advances in Machine Learning for Fiber Optic Sensor Applications. Adv. Intell. Syst..

[67] Krivosheev A.I., Barkov F.L., Konstantinov Y.A., Belokrylov M.E. (2022). State-of-the-Art Methods for Determining the Frequency Shift of Brillouin Scattering in Fiber-Optic Metrology and Sensing (Review). Instrum. Exp. Tech..

[68] Ge Z., Shen L., Zhao C., Wu H., Zhao Z., Tang M. (2022). Enabling variable high spatial resolution retrieval from a long pulse BOTDA sensor. IEEE Internet Things J..

[69] Karapanagiotis C., Wosniok A., Hicke K., Krebber K. (2021). Time-Efficient Convolutional Neural Network-Assisted Brillouin Optical Frequency Domain Analysis. Sensors.

[70] Karapanagiotis C., Hicke K., Krebber K. (2023). Machine learning assisted BOFDA for simultaneous temperature and strain sensing in a standard optical fiber. Opt. Express.

[71] Ruiz-Lombera R., Fuentes A., Rodriguez-Cobo L., Lopez-Higuera J.M., Mirapeix J. (2018). Simultaneous Temperature and Strain Discrimination in a Conventional BOTDA via Artificial Neural Networks. J. Light. Technol..

[72] Wang B.W., Wang L., Guo N., Zhao Z.Y., Yu C.Y., Lu C. (2019). Deep neural networks assisted BOTDA for simultaneous temperature and strain measurement with enhanced accuracy. Opt. Express.

[73] Yang G., Zeng K., Wang L., Tang M., Liu D. (2022). Integrated denoising and extraction of both temperature and strain based on a single CNN framework for a BOTDA sensing system. Opt. Express.

[74] Bao X., Webb D.J., Jackson D.A. (1994). Combined Distributed Temperature and Strain Sensor-Based on Brillouin Loss in an Optical-Fiber. Opt. Lett..

[75] Alahbabi M.N., Cho Y.T., Newson T.P. (2005). Simultaneous temperature and strain measurement with combined spontaneous Raman and Brillouin scattering. Opt. Lett..

[76] Coscetta A., Catalano E., Cerri E., Cennamo N., Zeni L., Minardo A. (2021). Hybrid Brillouin/Rayleigh sensor for multiparameter measurements in optical fibers. Opt. Express.

[77] Kee H.H., Lees G.P., Newson T.P. (2000). All-fiber system for simultaneous interrogation of distributed strain and temperature sensing by spontaneous Brillouin scattering. Opt. Lett..

[78] Kishida K., Yamauchi Y., Guzik A. (2014). Study of Optical Fibers Strain-Temperature Sensitivities Using Hybrid Brillouin-Rayleigh System. Photonic Sens..

[79] Liu X., Bao X. (2012). Brillouin Spectrum in LEAF and Simultaneous Temperature and Strain Measurement. J. Light. Technol..

[80] Peng J.Q., Lu Y.G., Zhang Z.L., Wu Z.N., Zhang Y.Y. (2021). Distributed Temperature and Strain Measurement Based on Brillouin Gain Spectrum and Brillouin Beat Spectrum. IEEE Photonic Technol. Lett..

[81] Zhang X., Liu S., Zhang J., Qiao L., Wang T., Gao S., Zhang M. (2021). Simultaneous Strain and Temperature Measurement Based on Chaotic Brillouin Optical Correlation-Domain Analysis in Large-Effective-Area Fibers. Photonic Sens..

[82] Zou L.F., Bao X.Y., Afshar V.S., Chen L. (2004). Dependence of the Brillouin frequency shift on strain and temperature in a photonic crystal fiber. Opt. Lett..

[83] Li Z.L., Yan L.S., Zhang X.P., Pan W. (2018). Temperature and Strain Discrimination in BOTDA Fiber Sensor by Utilizing Dispersion Compensating Fiber. IEEE Sens. J..

[84] Ekechukwu G.K., Sharma J. (2021). Well-scale demonstration of distributed pressure sensing using fiber-optic DAS and DTS. Sci. Rep..

[85] Hotate K., Hasegawa T. (2000). Measurement of Brillouin Gain Spectrum Distribution along an Optical Fiber Using a Correlation-Based Technique: Proposal, Experiment and Simulation (Special Issue on Optical Fiber Sensors). IEICE Trans. Electron..

[86] Hotate K. Recent achievements in BOCDA/BOCDR. Proceedings of the IEEE SENSORS 2014 Proceedings.

[87] Mizuno Y., Zou W., He Z., Hotate K. (2008). Proposal of Brillouin optical correlation-domain reflectometry (BOCDR). Opt. Express.

[88] Bao X., Brown A., DeMerchant M., Smith J. (1999). Characterization of the Brillouin-loss spectrum of single-mode fibers by use of very short (<10-ns) pulses. Opt. Lett..

[89] Liu Z., Ferrier G., Bao X., Zeng X., Yu Q., Kim A. (2003). Brillouin Scattering Based Distributed Fiber Optic Temperature Sensing for Fire Detection. Fire Saf. Sci..

[90] Farahani M.A., Castillo-Guerra E., Colpitts B.G. (2011). Accurate estimation of Brillouin frequency shift in Brillouin optical time domain analysis sensors using cross correlation. Opt. Lett..

[91] Farahani M.A., Castillo-Guerra E., Colpitts B.G. (2013). A Detailed Evaluation of the Correlation-Based Method Used for Estimation of the Brillouin Frequency Shift in BOTDA Sensors. IEEE Sens. J..

[92] Liang Y., Jiang J., Chen Y., Zhu R., Lu C., Wang Z. (2019). Optimized Feedforward Neural Network Training for Efficient Brillouin Frequency Shift Retrieval in Fiber. IEEE Access.

[93] Bishop C.M., Nasrabadi N.M. (2006). Pattern Recognition and Machine Learning.

[94] Ying X. (2019). An Overview of Overfitting and its Solutions. J. Phys. Conf. Ser..

[95] Prechelt L. (1998). Early Stopping—But when?. Neural Networks: Tricks of the Trade.

[96] Zhang Y., Yang Y. (2015). Cross-validation for selecting a model selection procedure. J. Econom..

[97] Lu C., Liang Y., Jia X., Fu Y., Liang J., Wang Z. (2020). Artificial Neural Network for Accurate Retrieval of Fiber Brillouin Frequency Shift with Non-Local Effects. IEEE Sens. J..

[98] Chen B., Su L., Zhang Z., Liu X., Dai T., Song M., Yu H., Wang Y., Yang J. (2022). Wavelet convolutional neural network for robust and fast temperature measurements in Brillouin optical time domain reflectometry. Opt. Express.

[99] Chang Y., Wu H., Zhao C., Shen L., Fu S., Tang M. (2020). Distributed Brillouin frequency shift extraction via a convolutional neural network. Photonics Res..

[100] Buber E., Diri B. Performance Analysis and CPU vs GPU Comparison for Deep Learning. Proceedings of the 2018 6th International Conference on Control Engineering & Information Technology (CEIT).

[101] Qi D., Li J., Guan X., Chan C.-K. (2022). Dynamic polarization-insensitive BOTDA in direct-detection OFDM with CNN-based BFS extraction. Opt. Express.

[102] Caceres J.N., Noda K., Zhu G., Lee H., Nakamura K., Mizuno Y. (2021). Spatial Resolution Enhancement of Brillouin Optical Correlation-Domain Reflectometry Using Convolutional Neural Network: Proof of Concept. IEEE Access.

[103] Lalam N., Venketeswaran A., Lu P., Buric M.P., Schröder H., Chen R.T. Probabilistic deep neural network based signal processing for Brillouin gain and phase spectrums of vector BOTDA system. Proceedings of the Optical Interconnects XXI.

[104] Soto M.A., Thévenaz L. (2013). Modeling and evaluating the performance of Brillouin distributed optical fiber sensors. Opt. Express.

[105] Meng X., Zhang D., Li H., Huang Y. (2022). Efficient two-stage strain/temperature measurement method for BOTDA system based on Bayesian uncertainty quantification. Measurement.

[106] Cortes C., Vapnik V. (1995). Support-vector networks. Mach. Learn..

[107] Yao Y., Mizuno Y. (2022). Dynamic strain measurement in Brillouin optical correlation-domain sensing facilitated by dimensionality reduction and support vector machine. Opt. Express.

[108] Zheng H., Xiao F., Sun S., Qin Y. (2022). Brillouin Frequency Shift Extraction Based on AdaBoost Algorithm. Sensors.

[109] Freund Y., Schapire R.E. (1997). A Decision-Theoretic Generalization of On-Line Learning and an Application to Boosting. J. Comput. Syst. Sci..

[110] Hastie T., Rosset S., Zhu J., Zou H. (2009). Multi-class AdaBoost. Stat. Interface.

[111] Quinlan J.R. Learning with Continuous Classes. Proceedings of the Australian Joint Conference on Artificial Intelligence.

[112] Dobra A., Gehrke J. SECRET: A scalable linear regression tree algorithm. Proceedings of the 8th ACM SIGKDD International Conference on Knowledge Discovery and Data Mining.

[113] Fix E., Hodges J.L. (1989). Discriminatory Analysis. Nonparametric Discrimination: Consistency Properties. Int. Stat. Rev. Rev. Int. Stat..

[114] Zheng H., Peng G.-D., He Z. Extraction of Brillouin frequency shift in Brillouin distributed fiber sensors by neighbors-based machine learning. Proceedings of the Advanced Sensor Systems and Applications X.

[115] Zheng H., Sun S., Qin Y., Xiao F., Dai C. (2022). Extraction of Brillouin frequency shift from Brillouin gain spectrum in Brillouin distributed fiber sensors using K nearest neighbor algorithm. Opt. Fiber Technol..

[116] Xiao F., Lv M., Li X. (2021). Fast Measurement of Brillouin Frequency Shift in Optical Fiber Based on a Novel Feedforward Neural Network. Photonics.

[117] Jolliffe I.T., Cadima J. (2016). Principal component analysis: A review and recent developments. Philos. Trans. R. Soc. A Math. Phys. Eng. Sci..

[118] Abdolrasol M.G.M., Hussain S.M.S., Ustun T.S., Sarker M.R., Hannan M.A., Mohamed R., Ali J.A., Mekhilef S., Milad A. (2021). Artificial Neural Networks Based Optimization Techniques: A Review. Electronics.

[119] Aszemi N.M., Dominic P. (2019). Hyperparameter optimization in convolutional neural network using genetic algorithms. Int. J. Adv. Comput. Sci. Appl..

[120] Yu T., Zhu H. (2020). Hyper-parameter optimization: A review of algorithms and applications. arXiv.

[121] Montavon G., Samek W., Müller K.-R. (2018). Methods for interpreting and understanding deep neural networks. Digit. Signal Process..

[122] Bansal M., Goyal A., Choudhary A. (2022). A comparative analysis of K-Nearest Neighbor, Genetic, Support Vector Machine, Decision Tree, and Long Short Term Memory algorithms in machine learning. Decis. Anal. J..

[123] Wang B., Guo N., Wang L., Yu C., Lu C. Denoising and Robust Temperature Extraction for BOTDA Systems based on Denoising Autoencoder and DNN. Proceedings of the 26th International Conference on Optical Fiber Sensors.

[124] Wang B., Guo N., Wang L., Yu C., Lu C. (2020). Robust and Fast Temperature Extraction for Brillouin Optical Time-Domain Analyzer by Using Denoising Autoencoder-Based Deep Neural Networks. IEEE Sens. J..

[125] Yang Y.-n., Dong Y., Yu K. SNR Improvement based on Attention-DNet for Brillouin Distributed Optical Fiber Sensors. Proceedings of the 2022 27th OptoElectronics and Communications Conference (OECC) and 2022 International Conference on Photonics in Switching and Computing (PSC).

[126] Wu H., Wan Y., Tang M., Chen Y., Zhao C., Liao R., Chang Y., Fu S., Shum P.P., Liu D. (2019). Real-Time Denoising of Brillouin Optical Time Domain Analyzer with High Data Fidelity Using Convolutional Neural Networks. J. Light. Technol..

[127] Zheng H., Yan Y., Wang Y., Shen X., Lu C. (2022). Deep Learning Enhanced Long-Range Fast BOTDA for Vibration Measurement. J. Light. Technol..

[128] Tian C., Xu Y., Li Z., Zuo W., Fei L., Liu H. (2020). Attention-guided CNN for image denoising. Neural Netw..

[129] Zheng M., Xu J., Shen Y., Tian C., Li J., Fei L., Zong M., Liu X. (2022). Attention-based CNNs for Image Classification: A Survey. J. Phys. Conf. Ser..

[130] Wu H., Wang L., Zhao Z., Guo N., Shu C., Lu C. (2018). Brillouin optical time domain analyzer sensors assisted by advanced image denoising techniques. Opt. Express.

[131] Hashemi M. (2019). Enlarging smaller images before inputting into convolutional neural network: Zero-padding vs. interpolation. J. Big Data.

[132] Azad A.K., Wang L., Guo N., Lu C., Tam H.Y. (2015). Temperature sensing in BOTDA system by using artificial neural network. Electron. Lett..

[133] Azad A.K., Wang L., Guo N., Tam H.Y., Lu C. (2016). Signal processing using artificial neural network for BOTDA sensor system. Opt. Express.

[134] Wang L., Wang B., Jin C., Guo N., Yu C., Lu C. Brillouin optical time domain analyzer enhanced by artificial/deep neural networks. Proceedings of the 2017 16th International Conference on Optical Communications and Networks (ICOCN).

[135] Wang J., Li Y., Liao J. (2019). Temperature extraction for Brillouin optical fiber sensing system based on extreme learning machine. Opt. Commun..

[136] Cao Z.Y., Guo N., Li M.H., Yu K.L., Gao K.Q. (2019). Back propagation neutral network based signal acquisition for Brillouin distributed optical fiber sensors. Opt. Express.

[137] Madaschi A., Morosi J., Brunero M., Boffi P. (2022). Enhanced Neural Network Implementation for Temperature Profile Extraction in Distributed Brillouin Scattering-Based Sensors. IEEE Sens. J..

[138] Li Y., Wang J. (2020). Optimized neural network for temperature extraction from Brillouin scattering spectra. Opt. Fiber Technol..

[139] Motil A., Hadar R., Sovran I., Tur M. (2014). Gain dependence of the linewidth of Brillouin amplification in optical fibers. Opt. Express.

[140] Wang B., Guo N., Khan F.N., Azad A.K., Wang L., Yu C., Lu C. Extraction of temperature distribution using deep neural networks for BOTDA sensing system. Proceedings of the 2017 Conference on Lasers and Electro-Optics Pacific Rim (CLEO-PR).

[141] Wang M.H., Sui Y., Zhou W.N., An X., Dong W. (2022). AIoT enabled resampling filter for temperature extraction of the Brillouin gain spectrum. Opt. Express.

[142] Wang M.H., Sui Y., Zhou W.N., Dong W., Zhang X.D. (2021). Sweep frequency method with variance weight probability for temperature extraction of the Brillouin gain spectrum based on an artificial neural network. Opt. Express.

[143] Zhang Y., Li Y., Cheng L., Yu L., Zhu H., Luo B., Zou X. Fast temperature extraction via Echo State Network for BOTDA sensors. Proceedings of the Asia Communications and Photonics Conference/International Conference on Information Photonics and Optical Communications 2020 (ACP/IPOC).

[144] Zhou H., Zhu H., Zhang Y., Huang M., Li G., Yang Y. Fast and accurate temperature extraction via general regression neural network for BOTDA sensors. Proceedings of the 12th International Conference on Information Optics and Photonics.

[145] Kumar S., Tiwari P., Zymbler M. (2019). Internet of Things is a revolutionary approach for future technology enhancement: A review. J. Big Data.

[146] Zhang Y., Yu L., Hu Z., Cheng L., Sui H., Zhu H., Li G., Luo B., Zou X., Yan L. (2021). Ultrafast and Accurate Temperature Extraction via Kernel Extreme Learning Machine for BOTDA Sensors. J. Light. Technol..

[147] Huang G., Huang G.-B., Song S., You K. (2015). Trends in extreme learning machines: A review. Neural Netw..

[148] Guang-Bin H., Hongming Z., Xiaojian D., Rui Z. (2012). Extreme Learning Machine for Regression and Multiclass Classification. IEEE Trans. Syst. Man Cybern. Part B.

[149] Wu H., Wang L., Guo N., Shu C., Lu C. (2017). Brillouin Optical Time-Domain Analyzer Assisted by Support Vector Machine for Ultrafast Temperature Extraction. J. Light. Technol..

[150] Wu H., Wang L., Zhao Z., Shu C., Lu C. (2018). Support Vector Machine based Differential Pulse-width Pair Brillouin Optical Time Domain Analyzer. IEEE Photonics J..

[151] Nordin N.D., Abdullah F., Zan M.S.D., A Bakar A.A., Krivosheev A.I., Barkov F.L., Konstantinov Y.A. (2022). Improving Prediction Accuracy and Extraction Precision of Frequency Shift from Low-SNR Brillouin Gain Spectra in Distributed Structural Health Monitoring. Sensors.

[152] Nordin N.D., Abdullah F., Zan M.S.D., Ismail A., Jamaludin M.Z., Bakar A.A.A. Fast temperature extraction approach for BOTDA using Generalized Linear Model. Proceedings of the 2020 IEEE 8th International Conference on Photonics (ICP).

[153] Nordin N.D., Zan M.S.D., Abdullah F. (2020). Generalized linear model for enhancing the temperature measurement performance in Brillouin optical time domain analysis fiber sensor. Opt. Fiber Technol..

[154] Nordin N.D., Zan M.S.D., Abdullah F. (2020). Comparative Analysis on the Deployment of Machine Learning Algorithms in the Distributed Brillouin Optical Time Domain Analysis (BOTDA) Fiber Sensor. Photonics.

[155] Murphy K.P. (2012). Machine Learning: A Probabilistic Perspective.

[156] Song Q., Zhang C., Tang G., Ansari F. (2020). Deep learning method for detection of structural microcracks by brillouin scattering based distributed optical fiber sensors. Smart Mater. Struct..

[157] Wei C., Deng Q., Yin Y., Yan M., Lu M., Deng K. (2022). A Machine Learning Study on Internal Force Characteristics of the Anti-Slide Pile Based on the DOFS-BOTDA Monitoring Technology. Sensors.

[158] Song Q., Yan G., Tang G., Ansari F. (2021). Robust principal component analysis and support vector machine for detection of microcracks with distributed optical fiber sensors. Mech. Syst. Signal Process..

[159] Zhang L., Shi B., Zhu H., Yu X., Wei G. (2020). A machine learning method for inclinometer lateral deflection calculation based on distributed strain sensing technology. Bull. Eng. Geol. Environ..

[160] Ruiz-Lombera R., Serrano J.M., Lopez-Higuera J.M. Automatic strain detection in a Brillouin Optical Time Domain sensor using Principal Component Analysis and Artificial Neural Networks. Proceedings of the IEEE SENSORS 2014 Proceedings.

[161] Lv T., Ye X., Zheng Y., Ge Z., Xu Z., Sun X. (2021). Error Estimation of BFS Extraction with Optimized Neural Network & Frequency Scanning Range. J. Light. Technol..

[162] Elshawi R., Wahab A., Barnawi A., Sakr S. (2021). DLBench: A comprehensive experimental evaluation of deep learning frameworks. Clust. Comput..

[163] Yao Y., Set S.Y., Yamashita S. Proposal of signal processing based on machine learning in Brillouin optical correlation domain analysis/ reflectometry. Proceedings of the 2017 22nd Microoptics Conference (MOC).

[164] Yao Y., Mizuno Y. (2021). Neural network-assisted signal processing in Brillouin optical correlation-domain sensing for potential high-speed implementation. Opt. Express.

[165] Chen X., Yu H., Huang W. A high accurate fitting algorithm for Brillouin scattering spectrum of distributed sensing systems based on LSSVM networks. Proceedings of the 2021 International Conference on Electronic Information Engineering and Computer Science (EIECS).

[166] Wan D., Shan L., Xi L., Xiao Z., Zhang Y.a., Yuan X., Zhang X., Zhang H. (2022). An improved lorentz fitting algorithm for BOTDR using SVM model to capture the main peak of power cumulative average data. Opt. Fiber Technol..

[167] Karapanagiotis C. Evaluation of the generalization performance of a CNN-assisted BOFDA system. Proceedings of the Sensors and Measuring Systems; 21st ITG/GMA-Symposium.

[168] Gyger F., Yang Z., Soto M.A., Yang F., Tow K.H., Thévenaz L. High Signal-to-Noise Ratio Stimulated Brillouin Scattering Gain Spectrum Measurement. Proceedings of the 26th International Conference on Optical Fiber Sensors.

[169] Rasmussen C.E., Williams C.K.I. (2005). Gaussian Processes for Machine Learning.

[170] Karapanagiotis C., Hicke K., Krebber K. Temperature and humidity discrimination in Brillouin distributed fiber optic sensing using machine learning algorithms. Proceedings of the Optical Sensing and Detection VII.

[171] Karapanagiotis C., Hicke K., Wosniok A., Krebber K. (2022). Distributed humidity fiber-optic sensor based on BOFDA using a simple machine learning approach. Opt. Express.

[172] Donoho D.L. (2006). Compressed sensing. IEEE Trans. Inf. Theory.

[173] Zhou D.-P., Peng W., Chen L., Bao X. (2018). Brillouin optical time-domain analysis via compressed sensing. Opt. Lett..

[174] Dong Y., Yang Y.-N., Azad A.K., Yang Z., Yu K., Zhao S. (2022). Compressed Sensing Based on K-SVD for Brillouin Optical Fiber Distributed Sensors. IEEE Sens. J..

[175] Zheng H., Yan Y., Zhao Z., Zhu T., Zhang J., Guo N., Lu C. (2021). Accelerated Fast BOTDA Assisted by Compressed Sensing and Image Denoising. IEEE Sens. J..

[176] Calderbank R. Compressed Learning: Universal Sparse Dimensionality Reduction and Learning in the Measurement Domain, *Preprint* 2009. https://www.semanticscholar.org/paper/Compressed-Learning-%3A-Universal-Sparse-Reduction-in-Calderbank/627c14fe9097d459b8fd47e8a901694198be9d5d#citing-papers.

